# Responsible, Inclusive Innovation and the Nano-Divide

**DOI:** 10.1007/s11569-016-0265-2

**Published:** 2016-06-09

**Authors:** Doris Schroeder, Sally Dalton-Brown, Benjamin Schrempf, David Kaplan

**Affiliations:** Centre for Professional Ethics, UCLAN College of Health, Brook 424, Preston, PR1 2HE England; Centre for Applied Philosophy and Public Ethics (CAPPE), CSU, Canberra, Australia; University of Melbourne, Parkville, VIC 3052 Australia; EA European Academy of Technology and Innovation Assessment GmbH, Wilhelmstraße 56, 53474 Bad Neuenahr-Ahrweiler, Germany; The School of Economics, New Building, Middle Campus, University of Cape Town, Private Bag X3, Rondebosch, 7701 South Africa

**Keywords:** Responsible research and innovation, Systems of innovation approaches, Inclusive innovation, Innovation governance systems, Nano-divide

## Abstract

Policy makers from around the world are trying to emulate successful innovation systems in order to support economic growth. At the same time, innovation governance systems are being put in place to ensure a better integration of stakeholder views into the research and development process. In Europe, one of the most prominent and newly emerging governance frameworks is called Responsible Research and Innovation (RRI). This article aims to substantiate the following points: (1) The concept of RRI and the concept of justice can be used to derive similar ethical positions on the nano-divide. (2) Given the ambitious policy aims of RRI (e.g. economic competitiveness enhancer), the concept may be better suited to push for ethical outcomes on access to nanotechnology and its products rather than debates based on justice issues alone. It may thus serve as a mediator concept between those who push solely for competitiveness considerations and those who push solely for justice considerations in nano-technology debates. (3) The descriptive, non-normative Systems of Innovation approaches (see below) should be linked into RRI debates to provide more evidence on whether the approach advocated to achieve responsible and ethical governance of research and innovation (R&I) can indeed deliver on competitiveness (in nano-technology and other fields).

## Introduction^1^

Academics, innovators and policy makers have for decades been interested in the dynamics that have made Silicon Valley a success (see also Table 1). Innovation and innovation systems are now becoming increasingly interesting to policy makers in order to achieve their economic and social goals.^2^ In Europe, “79 % of companies that introduced at least one innovation since 2011 experienced an increase of their turnover by more than 25 % by 2014” [1].

**Table 1:** Systems of innovation approaches

National Systems of Innovation (NSI)
Adopting a holistic view of innovation rather than focussing on isolated aspects of the process, the NSI concept emphasises the interaction of actors involved in innovation and analyses how these interactions are shaped by social, institutional and political factors [49]. NSI was remarkably successful in a short period of time and is now being used in academia and policy contexts [50]. It is often used as an analytical framework [51] for studying the differences between countries concerning their production and innovation systems [52].
Regional Systems of Innovation (RSI)
The NSI approach (above) assumes homogeneity within countries, but this is not necessarily the case. On many indicators (e.g. economic performance, poverty, R&D investment), countries can differ significantly within their own boundaries. As a result, researchers and scholars of innovation systems have developed a regionally based approach of innovation system thinking, with 'regions' usually referring to a geographical area within a country. In some instances, cross-border regions are also possible, the Saar Lorraine region being an example, which spreads across France and Germany and shows considerable collaboration in local economic affairs. The research focus in the Regional Systems of Innovation (RSI) concept therefore rests on the relationship between technology, innovation and industrial location [53]. This spatial concentration remains important for innovative activities, despite the argument that modern information and communication technologies would render spatial distances between communication partners unimportant [54]. Silicon Valley is normally used as the prime example of a region with great innovative potential.
Sectoral/Technological Systems of Innovation (S-TSI)
Unlike the innovation system approaches described above, which both rely on a spatial dimension to define their boundaries, the sectoral/technological innovation system approaches adopt either a certain technology (spanning multiple sectors) or the sector in which it is used (including various technologies) as their system boundary. The notion that particular sectors have different technological trajectories was first spelt out by Dick Pavitt [55]. The concept of sectoral innovation systems was further developed by Malerba [56], whereas the development of the technological approach can be traced back to Carlsson and Stankiewicz [57]. Both concepts are less developed than the NSI and the RSI approaches and have a smaller overall impact. In both sectoral and technological systems of innovation, links between firms and other organisations are portrayed as occurring as a result of the technological interdependence of their knowledge [58].

As a result, policy makers from around the world are trying to emulate successful innovation systems in order to support economic growth. At the same time and following negative societal responses to genetic modification around the world, innovation *governance* systems are being put in place to ensure a better integration of stakeholder views into the research and development process. In Europe, one of the most prominent and newly emerging governance frameworks is called Responsible Research and Innovation (RRI) [2].

This article is in four parts. The first part provides background, definitions and clarifications about the terms innovation, innovation systems and responsible research and innovation. The second part will consider the question of the nano-divide with reference to RRI. The third part will introduce the concept of inclusive innovation to bridge the gap between innovation systems and RRI. Finally, the conclusion will substantiate the following three points:

1. The concept of RRI and the concept of justice can be used to derive similar ethical positions on the nano-divide.^3^
2. Given the ambitious policy aims of RRI (e.g. economic competitiveness enhancer), the concept may be better suited to push for ethical outcomes on access to nano-technology and its products rather than debates based on justice issues alone. It may thus serve as a mediator concept between those who push solely for competitiveness considerations and those who push solely for justice considerations in nano-technology debates.
3. The descriptive, non-normative Systems of Innovation approaches (see below) should be linked into RRI debates to provide more evidence on whether the approach advocated to achieve responsible and ethical governance of research and innovation (R&I) can indeed deliver on competitiveness (in nano-technology and other fields).

## Innovation, Innovation Systems and Responsible Research and Innovation

Innovation has been defined as follows:

Innovation is an activity or process which may lead to previously unknown designs pertaining either to the physical world (e.g. designs of buildings and infrastructure), the conceptual world (e.g. conceptual frameworks, mathematics, logic, theory, software), the institutional world (social and legal institutions, procedures and organisation) or combinations of these, which—when implemented—expand the set of relevant feasible options for action, either physical or cognitive [3].

Innovation is widely regarded as the key ingredient to national economic success. For instance, China, the country which was most successful worldwide in terms of economic growth in 2013 (7.7 %) [4], recently launched structural adjustment policies to move from manufacturing growth towards a knowledge and innovation economy. In 2012, the 18th National Congress of the Communist Party of China proposed a reform of the science and technology system to improve the potential for innovations across all sectors [5].

As innovation has become central to economic success, policy makers and researchers are increasingly interested in understanding what factors enhance innovation. A range of descriptors have emerged for fields that examine the innovation process from knowledge creation to commercialisation (e.g. innovation studies, science studies, science and innovation studies, science and technology studies). One of the fields’ most prominent outputs is the Systems of Innovation approach. The three main Systems of Innovation approaches are the National Systems of Innovation approach (NSI), the Regional Systems of Innovation (RSI) approach and the Sectoral/Technological Innovation Systems approach (S-TSI; see Table 1).

Apart from the distinctions given in the above table, all three Systems of Innovation (SI) approaches share certain characteristics. They all place great emphasis on the learning process [6], in which all actors involved (e.g. firms, consumers, universities, public organisations) experience 'learning-by-doing' or learn from each other by exchanging knowledge. Systems of innovation are always defined as complex systems [7], stressing their non-linear, systemic, interactive and evolutionary character [8, 9]. Furthermore, the performance of all SI approaches is analysed in a similar way, namely through the ex-post, historical analyses of economic or innovative activity and knowledge diffusion [10]. Such analyses are holistic and interdisciplinary, bringing together scholars and analysts from various disciplines to account for the many and complex interactions in the system [6].

The attractiveness of SI approaches for policy makers is the fact that they can draw attention to strengths and weaknesses in the innovation system [11]. However, it is important to emphasise that SI approaches aim to be purely descriptive. These approaches investigate which actors belong to the system, which networks are formed, what the boundaries of the system are, which knowledge is generated and which internal dynamics can be observed [12]. In other words, whilst SI research might describe normative behaviour when found in the innovation process, it tries not by itself to generate any normative conclusions. For instance, policy makers could use research from innovation studies in making funding or tax incentive decisions, based on, for example, the reasoning that successful innovation systems have the potential to reduce unemployment and thereby poverty. For instance, a scheme that provides tax incentives to innovators who are most likely to be successful according to SI research could be defended with reference to job creation and its potential for poverty reduction.

However, innovation is not only seen as a desirable driver of economic growth and prosperity. It can also be highly contentious and even adversarial, particularly in the context of new and emerging technologies, where significant risks for humankind, the environment, local populations, and researchers can occur. It is in this context that the field of Technology Assessment (TA) has been developed [13] and enhanced [14] as a key mechanism to govern science and innovation. However, by contrast to the emergence of TA, which was highly expert-driven, newer concepts of innovation governance aim to involve more stakeholders in the innovation process.

In recent years, the new governance framework of RRI or Responsible Innovation (RI) has become prominent in Europe. The European Commission is highly active in supporting models which govern research and innovation in such a way that societal concerns and interests are taken into account. The ‘Science with and for Society’ (SWAFS) programme has produced one of the most influential RRI definitions in Europe.

RRI is an inclusive approach to research and innovation (R&I), to ensure that societal actors work together during the whole research and innovation process. It aims to better align both the process and outcomes of R&I, with the values, needs and expectations of European society. In general terms, RRI implies anticipating and assessing potential implications and societal expectations with regard to research and innovation [2].

The European Commission, which promotes RRI, is also the organisation which drives European competitiveness.

The European Commission places great emphasis on competitiveness, given its importance in creating jobs and growth in Europe. It works to mainstream industry-related competitiveness concerns across all policy areas [15].

It is noteworthy that RRI has been linked to increased economic competitiveness in a report published by the European Commission.

The consideration of ethical and societal aspects in the research and innovation process can lead to an increased quality of research, more successful products and therefore an increased competitiveness [3].

The European Commission has also issued a range of funding calls to provide more evidence on the link between RRI and increased economic competitiveness. For instance, the call “Responsible Research and Innovation in an industrial context”

aims to contribute towards the innovation and competiveness objectives of the Innovation Union and to enhanced ‘mainstreaming’ and standardisation of RRI and CSR processes at the EU and global level.^4^

Hence, the approach to research and innovation promoted by the European Commission through their understanding of RRI is closely linked to economic competitiveness.

Another RRI definition developed in Europe by Rene von Schomberg defines RRI as a

[T]ransparent, interactive process by which societal actors and innovators become mutually responsive to each other with a view on the (ethical) acceptability, sustainability and societal desirability of the innovation process and its marketable products (in order to allow a proper embedding of scientific and technological advances in our society) [16].

Amongst academics, the most prominent definition of RRI, which was agreed by European and US authors in a joint publication, is “Responsible innovation is a collective commitment of care for the future through responsive stewardship of science and innovation in the present” [17]. In implementing responsive stewardship, the following four RRI dimensions are necessary, according to the authors: anticipation, reflection, deliberation and responsiveness.

What all three definitions of R(R)I have in common is that they demand the involvement of a variety of societal actors in the innovation process. They also stress the importance of care, responsiveness and aligning innovation with societal values and needs.

In this article, we will focus on one essential element from each definition and link them to nano-technology. From the SWAFS definition advocated by the European Commission, we will focus on societal needs, which we will interpret as global societal needs.

It might be asked why we would jump from the “needs… of European society” to the needs of global society. There are many reasons for doing so, including a large literature on cosmopolitanism, but we shall focus on two reasons that can be specifically related to nano-technology.

Considering *only* the needs of societies at a national or regional level within innovation governance frameworks disregards the responsibilities Northern states have, historically and currently, for the societal needs of Southern states. Thomas Pogge has successfully illustrated a network of obligations from North to South with concrete examples, which show that these duties do not derive from obligations of benevolence or charity [18]. Intellectual property rights are one instance where innovation governance frameworks systematically favour high income over low- and middle-income countries [19]. Hence, if innovation governance frameworks that structurally favour one set of agents, including nano-technology innovators, are already in place globally (such as the IPR system), one cannot reasonably limit the extension of another innovation governance framework (RRI) to favour the same set of agents yet again, by limiting it to only regional (European) significance.

More specifically, and in relation to nano-technology, it has been argued that “Nano-technology can be harnessed to address some of the world’s most critical development problems, … [including] challenges faced by the 5 billion people living in the developing world” [20]. Indeed, in a globalised world, one cannot reasonably ignore the potential of a technology for impacting on the lives of the most vulnerable people on Earth, by restricting a discussion on its development to the needs of European society. Hence, whilst we use one element from the SWAFS definition of RRI (needs), we believe that its restricted focus on Europe cannot be justified, and we therefore expand the scope of our discussion to be global.

From the von Schomberg definition, we will focus on societal desirability, which we define as follows: “An innovation is societally desirable, if it can benefit all human beings without discrimination”. One could ask why we interpret ‘societal desirability’ to relate to innovations that can benefit all human beings without discrimination. Is that not too demanding? Societal desirability is an inadequately defined term in the literature. Its strong advocate, Rene von Schomberg, has linked it to the right impacts and outcomes of research [16]. Trying to answer what such impacts and outcomes would be, he links societal desirability to the grand challenges of humankind, for instance, climate change, public health, pandemics and security [16].

That is one possible answer, but it is both more demanding than our suggestion and also restricts the number of societally desirable innovations even further. Our interpretation of societal desirability does at least leave the door open for innovations that have the potential to benefit all of humanity without addressing the grand challenges. For instance, Information and Communication (ICT) tools to improve pre-school learning have the potential to benefit all human beings without relating to a grand challenge of humanity. Hence, our take on the societal desirability criterion of RRI is less ambitious than Rene von Schomberg’s, and we therefore assume that taking it forward in this article is reasonably justifiable.

This is not to say however that *all* innovation has to be targeted in such a way that *all* of humankind must *always* potentially benefit from it. We believe that von Schomberg’s societal desirability criterion simply has the potential to widen the sphere of potential beneficiaries of research and innovation and that such an extension of the concept will distinguish highly responsible from less responsible innovation.

One could also ask whether societal desirability is not the same as ethical acceptability. Obviously, it is ethically acceptable for all of humankind to benefit from innovations without discrimination. And, after all, ethics is the study of *all* moral principles and systems as well as the study of right and wrong conduct. Hence, any researcher and innovator responsibilities could fall under this heading. However, to understand what RRI implies, it is important to divide it into more easily understandable pieces. Even though the above broad understanding of ethical acceptability is plausible, we shall use the term here in a more limited manner. For the purposes of this paper, ethical acceptability will be equated with the demand to not fundamentally transgress societal values, which includes compliance with research ethics (e.g. do not exploit research participants). This means it is understood in a limiting way, linked to “doing no harm”. By contrast, societal desirability is understood as “doing good”. For instance, Article 15 (1) of the UNESCO Declaration of Bioethics and Human Rights requires that

Benefits resulting from any scientific research and its applications should be shared with society as a whole and within the international community, in particular with developing countries [21].

This relates to societal desirability, whilst most other articles in the declaration relate more directly to ethical acceptability (e.g. Article 4 on harm, Article 6 on consent).

Thirdly, we will focus on responsiveness, which Owen et al. interpret as

[U]sing a ‘collective process of reflexivity to both set the direction and influence the subsequent trajectory and pace of innovation, through effective mechanisms of participatory and anticipatory governance. This should be an iterative, inclusive and open process of adaptive learning, with dynamic capability’ [17].

One might wonder what an iterative, inclusive and open process of adaptive learning with dynamic capability would look like; how expensive it would be; and how successful it could be. However, such questions are related directly to critiques of the definitions themselves. Here, we shall simply examine their application in our nano-technology case study.

Our first two RRI elements (societal needs, societal desirability) are therefore *outcome* or output based. The innovation output is intended to relate to global societal needs and have the potential to benefit all human beings without discrimination. The third RRI element we are considering here, responsiveness, describes the ideal *process* by which to define what counts as a global societal need and what counts as benefitting humankind without discrimination.

## The Nano-Divide; Societal Needs, Societal Desirability and Responsiveness

Some people predict that nano-technology will be at the centre of the next significant innovation wave with its ‘revolutionary’ potential in terms of its impact on industrial production [22]. One of the main ethical criticisms of nano-technology is summarised in the term ‘nano-divide’, which has been used since at least 2001 [23]. It refers to differing access to nano-technology between low-, middle- and high-income countries. A rather more politically loaded term is ‘nano-apartheid’ [24], which gives an indication of the emotive nature of this ethical debate.

The term nano-divide can be understood in two main ways, according to Cozzens and Wetmore [25]. First, the ‘nano-innovation divide’, which refers to “inequity based on where knowledge is developed and retained and a country’s capacity to engage in these two processes”, and second, the ‘nano-orientation divide’, which refers to “inequity based on the areas in which nano-technology research is targeted”. Hence, one use of the term relates to the capacity for nano-technology development and commercialisation, whilst the other is about the distribution of benefits from its use.

Societal needs, societal desirability (understood as the potential to benefit all human beings without discrimination) and responsiveness are the RRI criteria we have selected for a discussion of the nano-divide. The first two RRI criteria we specified focus solely on Cozzens and Wetmore’s second understanding of the nano-divide, namely the *targets* of nano-technology. In other words, societal needs and the potential of innovation to benefit all human beings without discrimination are linked to the benefits of the use of nano-technology. Is research targeted at clean water or improved cosmetics? These criteria are not directly linked to the capacity to undertake nano-technology research.

Responsiveness, on the other hand, would be required in relation to both understandings of the nano-divide. First, some technologies might not be acceptable to the public in the first place, in which case the required collective reflection would focus on the question of “what futures do we collectively want science and innovation to bring about and on what values are these based?” [17] Second, to give direction to individual innovations requires the iterative, inclusive and open process Owen et al. envisage when they define responsiveness in innovation. Hence, the three criteria from RRI definitions we have chosen have the potential to cover the same ground as the debates Cozzens and Wetmore have surveyed to develop their distinction.

Both understandings of the nano-divide have already been discussed widely in nano-ethic circles. For instance, Celine Kermisch has asked: given that nano-technology is likely to offer advances in areas of significant benefit to low- and middle-income countries such as new medicines (better HIV retrovirals is one of her examples), is there a moral obligation to share such life-enhancing technologies? [26] Note, she does not ask whether to share the outputs of nano-technology innovation but the technology itself. In other words, she does not talk about providing access to medicines but about sharing the technology to develop them.

At the same time, when the nano-industry itself advertises potential applications, the focus is on the sharing of innovation outcomes rather than technology sharing. For instance, a report from the Nanotechnology Industries Association indicates that use of nano-technology could transform the remote and poverty-stricken areas of the world with innovations such as water nano-filters, ‘labs on a chip’ that could assist rural doctors, cheaper drugs, batteries that utilise nano-technology for longer life, improved pesticides and fertilisers, environmental nano-cleansing of contaminated ground, lightweight construction materials that can be transported more cheaply and better food storage packaging [27].

The gap between real-life innovations and aspirations to develop innovations to assist the under-privileged is often the target of criticism. For instance, it is argued that to date, most nano-technology innovations have been directed at high-income world products that are more profitable, such as self-cleaning glass, tennis balls and cosmetics, and thus, nano-technology has been condemned for its potential to advance Northern consumerism whilst creating few products aimed at benefitting the poor [28]. In this context, Geoffrey Hunt asks “can we at last… make an international cooperative effort to put nano-technological developments at the service of human and ecological welfare, or will it be primarily nano-technology for more over-consumption?” [29].

The combination of high-tech innovation potential with possibly enormous societal, medical and environmental impact has always offered an uneasy dilemma for society, and more specifically policy makers, about whether profitability or tackling world societal challenges might be more important [30].

When approaching the nano-divide from a distributive justice point of view,^5^ it has been argued that access to nano-technology might come to be seen as a right of citizenship, in the same way as access to medical care [31]. “If nano-technology really is as revolutionary as proponents suggest, then both justice and a concern for the stability of any global political order require that we negotiate the challenges of the nano-divides” [31].

This summarises the discussion of the nano-divide from a philosophical perspective. But, is there anything instructive one can learn from approaching the nano-divide from an RRI angle? We want to focus on two points.

First, RRI is a research and innovation governance framework on the rise in Europe, developed—amongst others—by the European Commission, the institution which works to improve economic competitiveness, as noted above. Hence, if the same institution was to push both for profitability *and* addressing societal challenges through innovation focusing on societal needs, the audience reached with information about the nano-divide would probably be larger. In other words, the European Commission might command a larger audience of listeners and readers, and have more influence, than the authors of philosophical papers and books. For instance, one could venture that industry is more interested in pronouncements from the European Commission than the arguments of distributive justice philosophers. Of course, one has to note that the European Commission’s own definition of RRI focuses solely on the “needs and expectations of *European* society” [2] (our emphasis). For the reasons given above, however, this is unjustifiably Eurocentric in a world where innovation governance frameworks have historically been rolled out to the detriment of low- and middle-income countries and to the benefit of Europe (and other high-income regions). Hence, RRI combined with some basic justice considerations^6^ could provide an angle on the nano-divide that comes from an institution known for its focus on economic competitiveness.

Second, if one discusses competitiveness, the nano-divide and RRI in the same breath, one is situated more harmoniously in the centre rather than at either end of another important debate, the benefits and challenges of patents. In terms of a sole focus on competitiveness from a high-income country perspective, one would argue that patents rightly bar entry to competitors in order to “provide the innovator firm with an opportunity to price above the marginal cost and thereby recoup R&D expense” [32]. In terms of a sole focus on the nano-divide, one would stress the access problems of low- and middle-income economies and related unmet human needs. RRI could be seen as a mediator concept here, which tries to combine a concern for competitiveness with a concern for the satisfaction of needs.

The trickle-down effect has often been used to try and marry the concerns of profitability and societal desirability, arguing that what initially benefits the rich will become available to poorer populations later. In the context of nano-technology, it is “likely that many of the benefits nano-technology can provide to the developing world will be delayed by at least a generation or more—the 20-year term of a patent” [33]. Kathy Wetter argues that researchers and innovators in the South are likely to find that participation in the proprietary nano-technology revolution is “highly restricted by patent tollbooths, obliging them to pay royalties and licensing fees to gain access” [34]. However, a survey of global nano-health patents filed between 1975 and 2004 showed that China owned 20 % of internationally filed patents, second only to the USA (33 %) and ahead of Germany with 13 % [35].

An example of where nano-technology research takes place in a lower middle-income country focused on a societal challenge is in South Africa, where tuberculosis (TB) is the leading cause of death. Approximately 80 % of the population have latent TB, and the incidence of drug-resistant TB is also a major concern [36]. TB is curable but only with long drug courses (6 months for standard TB and 2 years for drug-resistant TB) that are well supervised. Researchers in South Africa are therefore working on a way to incorporate tuberculosis drugs into nano-particles so that they are released slowly into a patient’s bloodstream, raising the possibility that a regime of daily pills could be replaced by a single weekly dose. Despite the expense of development, “the potential advantages of the technology make its pursuit worthwhile. If TB treatment is reduced to a once-a-week dose, the overall costs, both of the drugs and of employing healthcare staff, could be significantly reduced” [37].

A 2013 Nanotechnology Industries Association Report is optimistic about the resolution of the nano-divide, claiming that

Nano-technology is still in its infancy and will take time to deliver on its promises. The developing world will also need time to appropriate the technology so as to make the most out of it and to boost its economies. Global inequality shall not be widened by nano-technology in and of itself; nevertheless, nano-technology offers a positive influence in reducing the divide between the rich and the poor by providing new approaches to tackle the challenges faced by the developing world, and as such, its impact will vary according to how it is implemented [27].

Discussing the nano-divide in the context of RRI might broaden the debate by moving from discussions about pure justice to larger RRI discussion circles. Yet, the debate could be broadened even more if innovation systems could be included within it, as these are of prime interest to policy makers and are allegedly descriptive or non-normative.

## The Nano-Divide, Innovation Systems and Inclusive Innovation

As noted above, the (SI) approach is the predominant approach by which researchers and policy makers try to make sense of successful innovations which emerge from a whole network of enabling conditions. SI approaches aim to be purely descriptive or at least without explicit normative elements. By contrast, the nano-divide is a discussion almost exclusively about normative elements. Who *should* have access to the technology and the outputs of the technology, given that the market will not secure coverage for all those who need it?^7^ In this regard, the two debates stand at different poles of a spectrum. How could they be combined?

SI research is used by policy makers to steer the system so that innovation can flourish. In this regard, we have a link to RRI. RRI is an approach promoted by policy makers to guide innovation once it is happening; hence, one step after SI research helps to analyse the system. However, there is a third area of research interest that could fit into these debates, inclusive innovation. Inclusive innovation combines elements from innovation research with a strong, explicit normative element.

Following the Millennium Development Goals^8^ [38], which sought to improve the economic and social position of the poor, there has been an upsurge of interest in ‘pro-poor’ or ‘inclusive’ growth. Since innovation plays a key role in growth and in determining the character of growth and the distribution of its benefits, increasing attention has been paid to innovation policies and practices that have the potential to assist the poor.

The term ‘inclusive innovation’ is now very widely employed. International agencies such as the World Bank have embraced the term, and the United Nations Development Programme (UNDP) maintains an International Policy Centre for Inclusive Growth headquartered in Brasilia, Brazil. A large number of governments, notably in low- and middle-income countries—for example, India and Thailand [39]—have developed or are in the process of developing explicit policies focused on inclusive innovation. The Indian government characterised the 2010–2020 decade as the “Decade of Innovation” and created the National Innovation Council in 2011, with a specific brief to promote inclusive innovation at the national and state levels [40]. China’s 12th Five Year Plan (2011–2015) shifts the focus from pursuing economic growth to sharing the benefits of development with all people, and innovation has a key role to play in this. Research organisations such as the Global Research Alliance have placed inclusive innovation at the centre of their objectives [41].

However, there is as yet no agreed definition of the term inclusive innovation, and indeed, a variety of similar terms are employed in different contexts. These terms include pro-poor innovation, below the radar innovation, bottom of the pyramid innovation, grassroot innovation and Jugaad or frugal innovation [42, 43].

What all of these terms have in common is that they refer to the production and delivery of innovative solutions to the problems of the poorest and most marginalised communities and income groups. Some definitions require that the poor are, in some way, actively engaged in the innovation process itself. A broad definition would therefore be “inclusive innovation is the means by which new goods and services are developed for and/or by the billions living on the lowest incomes” [44].

It is possible to conceive of a number of different levels at which ‘inclusivity’ could potentially operate.

- The poor being engaged in the definition of the problems to be addressed such that the innovation is relevant to the needs of the poor;
- The poor being actively engaged in some manner in the development and application of innovative solutions to their problems;
- The poor being engaged in the adoption, assimilation and diffusion of innovative solutions to their problems;
- The poor being engaged in the impact of innovation, such that the innovation outputs maximise the consumption and/or incomes of the poor [44].

Some protagonists and advocates of inclusive innovation look to the inclusion of poorer people as active participants in the processes of innovation [45]. This perspective also defines inclusive innovation in terms of the innovation *process* and not merely in terms of the *outcome*. It seeks innovative activity that, in some way, has the potential to enhance the capacities of poor people. As a result, they would not merely be passive recipients of innovation but instead be actively engaged. The active engagement of the poor in the innovation process finds its strongest expression in grassroot or community innovation movements. “Grassroot innovation movements seek innovation processes that are socially inclusive towards local communities in terms of the knowledge, processes and outcomes involved” [46].

At first sight, it looks as though RRI and inclusive innovation differ significantly. Inclusive innovation focuses almost exclusively on the needs of the poor, for instance, as beneficiaries of innovation or as co-innovators. By contrast, the term inclusive within RRI definitions has no pro-poor focus and is only one amongst many criteria that determine whether research and innovation is undertaken responsibly. For instance, the six key action points agreed by the European Commission’s SWAFS’ unit to determine whether research and innovation is undertaken responsibly are governance, public engagement, gender equality, science education, open access/open science and ethics [2]. Only one SWAFS report has added other action points, namely sustainability, and social justice/inclusion [47]. Hence, ‘inclusion’ plays a much smaller role in RRI than it does in inclusive innovation.

However, both inclusive innovation and RRI mirror the above conceptualisation of the nano-divide between innovation *for* and innovation *with* end-users. Inclusive innovation requires the development of new goods and services *for* the billions living on the lowest incomes whilst also requiring engagement *with* the poor in the development, adoption, assimilation and diffusion of innovative solutions for their problems. For RRI, the targeting of innovation at societal needs and the inclusion of end-users in innovation processes aims to achieve a better alignment of both the process *and* the outcomes of research and innovation with the needs of all of society.

If one tried to bring ‘inclusive innovation’ closer to RRI, one could argue that the term inclusion would require that *all* segments of society benefit from and influence innovation. ‘Pro-poor’ innovation, on the other hand, is a less suitable concept, as it focuses more clearly on one segment of the population only. Whilst one can provide strong arguments for an *exclusive* focus on the poor, as—for instance—John Rawls did with the difference principle^9^ in his ground-breaking ‘A Theory of Justice’ [48], RRI definitions focus on the entire population. For instance, the European Commission defines RRI as “an inclusive approach to research and innovation”, as noted above, not one that is focused on the under-privileged. Inclusive innovation is then not about the exclusion of richer populations from innovation and its benefits but about the broadening of the network positively impacted by innovation to include all.

Hence, RRI and inclusive innovation can be linked straightforwardly. However, what about the elusive link to the descriptive-only innovation systems approaches? From the brief account given above, we know that innovation system analysts try to find out, amongst other things, who is involved with which activities in innovation systems. As such, if policies such as RRI or inclusive innovation are successfully realised, innovation system analysts will find larger, more diverse networks, which also include new actors within their systems. If more population groups and more diverse end-user groups are included, for instance, the innovation system will grow. The important task for Systems of Innovation analysts is then to be sensitive to the pronouncement of RRI and inclusive innovation and its individual components (e.g. societal engagement, gender equality) in order to ascertain whether they improve innovation systems or not. If they can find convincing evidence, this would in turn validate the European Commission’s SWAFS’ unit claim that RRI is conducive to economic competitiveness.

Innovation system analysts are important contributors to the RRI debate, as they are best placed to ascertain whether policy makers’ claims are valid. For instance, does the RRI governance framework indeed increase economic competitiveness? That is a very broad claim. Broken down into smaller claims would probably be more meaningful. Research from innovation system analysts would then answer questions such as: In which sectors is RRI likely to lead to enhanced economic competitiveness, if any? In which regions is RRI likely to lead to enhanced economic competitiveness, if any? Which role do certain actors play within the innovation system with regards to RRI?

As a relatively new concept, RRI needs statistical and case study support for the broad claims it makes, in particular for being able to marry increased social justice (e.g. gender equality, engagement, open access) with increased economic competitiveness.^10^ Innovation system analysts are well placed to provide such data when assessing how responsible research and innovation case studies can be linked to existing approaches (see also Table 1). Likewise, proponents of inclusive innovation need statistical and case study support to ensure that their normative aims are reached. A possible next step for SI analysts in assisting the further development of RRI or inclusive innovation would be to co-develop relevant indicators that could be used, for instance, in computer-simulated models of innovation systems and innovation networks.

## Conclusion

RRI and inclusive innovation inject moral values into innovation governance systems. Although there is no specific mention of justice in RRI, the implicit framing around justice concepts becomes obvious when one compares nano-divide debates from an RRI perspective and from a traditional philosophical justice perspective. Both approaches can arrive at very similar results. It is undesirable if a technology which has a major potential to improve the lives of the poorest people remains inaccessible to those countries and end-users who need them. Hence, to push for better access to nano-technology and its innovative outputs, one could use the concept of RRI, enhanced with some arguments from the philosophical justice literature. Given RRI’s pedigree in Europe (namely its development from within the European Commission and therefore its close relationship to economic competitiveness efforts), using RRI pragmatically to push for broader access to nano-technology and its innovations may give better results than using justice arguments alone.

## Footnotes

1 Thanks for the helpful comments from two anonymous referees.

2 For instance, a reduction in unemployment through economic growth.

3 Considerations of justice are not often discussed in the context of RRI. An example exception is [38].

4 http://ec.europa.eu/research/participants/portal/desktop/en/opportunities/h2020/topics/18099-garri-2-2015.html

5 This article does not provide the scope to discuss the concept of justice in detail. For interpretations of different conceptions of justice relevant to international governance frameworks, see [59]. Distributive justice is relevant here, as it covers questions of access to technology. A typical distributive justice question is does international distributive justice require the sharing of advanced technologies with less technologically advanced communities in order to improve their prospects? Questions of corrective justice could be relevant where the less technologically advanced communities have been harmed by the more technologically advanced communities.

6 A basic justice consideration here would be the Kantian demand not to violate perfect duties. See governance changes to the international intellectual property rights system [19] that predictably led to higher morbidity/mortality in low- and middle-income countries.

7 In this article, we do not deal with the normative question of whether the technology should be used in the first place.

8 On 25 September 2015, the Millennium Goals were superseded by the UN Sustainable Development Goals. The new agenda consists of 17 goals designed to end poverty and hunger by 2030 [60].

9 The difference principle is based on a simple idea. Given that efforts to achieve full equality in society (which might be regarded as the most just outcome) will invariably lead to systematic and chronic inefficiencies, some inequalities will be allowed but only if they lead to advantages for the least well off. The difference principle would therefore allow higher salaries for surgeons if it could be shown that their services would not otherwise be available to the least well off.

10 Thanks to John Weckert and Mary Walker for pointing out this tension.
